# 

**Published:** 1894-11

**Authors:** 


### CONTENTS—NOVEMBER, 1894.—VOL. X., NO. 5.

ORIGINAL COMMUNICATIONS—
The Increase of Sexual Debility in Males. James Orr, M. D., Terrell 207
Puerperal Eclampsia. Robert Morris, M. D., Houston . . . . . . 213
Cystitis. E. T. Cook, M. D., Houston . . . . . . . . . . . . . . . 218
CORRESPONDENCE—
Medical Organization in Texas . . . . . . . . . . . . . . . . . 221
The New Mexican Remedy for Dysentery . . . . . . . . . . . . . 223
The Transactions: A Correction . . . . . . . . . . . . . . . . . 224
We Need a Better Medical Law . . . . . . . . . . . . . . . . . . 225
CURRENT MEDICAL LITERATURE—
Department of Therapeutics. David Cerna, M. D., Galveston . . . 226
Gynecological Notes. T. Flavin, M. D., Galveston . . . . . . . . 230
Notes on Dermatology. Isadore Dyer, M. D., New Orleans . . . . . 232
SOCIETY NOTES—
Northwest Texas Medical Association—Second Semi-Annual Meeting 233
Medico-Legal Society and Section on Medico-Legal Surgery . . . . 237
ABSTRACTS AND SELECTIONS—
Emasculation of Masturbators—Is It Justifiable? . . . . . . . . 239
The American Public Health Association . . . . . . . . . . . . . 244
EDITORIAL—
Rally to the Standard . . . . . . . . . . . . . . . . . . . . . 247
The Transactions—The Association, etc . . . . . . . . . . . . . 249
MEDICAL NEWS AND MISCELLANY—
Numerous Paragraphs . . . . . . . . . . . . . . . . . . . . . . 250
Hygiene of the Young in Schools . . . . . . . . . . . . . . . . 256
BOOK NOTICES— . . . . . . . . . . . . . . . . . . . . . . . . . 258
PUBLISHERS' NOTES . . . . . . . . . . . . . . . . . . . . . . . 263

